# Hpgd affects the progression of hypoxic pulmonary hypertension by regulating vascular remodeling

**DOI:** 10.1186/s12890-023-02401-y

**Published:** 2023-04-13

**Authors:** Meng He, Kelong Tao, Min Xiang, Jian Sun

**Affiliations:** 1 Department of Respiratory and Critical Care Medicine, Shaoxing People’s Hospital, No. 568 Zhongxing North Road, Shaoxing, Zhejiang Province 312000 China; 2 Department of Gastrointestinal Surgery, Shaoxing People’s Hospital, No. 568 Zhongxing North Road, Shaoxing, Zhejiang Province 312000 China

**Keywords:** Hypoxic pulmonary hypertension, Trajectory analysis, WGCNA, Angiogenesis, Hpgd

## Abstract

**Background:**

Hypoxic pulmonary hypertension (HPH) is a syndrome of abnormally elevated pulmonary artery pressure, and it is mostly caused by vasoconstriction and remodeling of the pulmonary artery induced by long-term chronic hypoxia. There is a high incidence of HPH, a short survival time of the patients, but currently no effective treatments.

**Methods:**

In this study, HPH-related single cell sequencing (scRNA-seq) and bulk RNA sequencing (RNA-seq) data were downloaded from the public database of Gene Expression Omnibus (GEO) for bioinformatics analysis in order to find out genes with important regulatory roles in the development of HPH. 523 key genes were identified through cell subpopulation identification and trajectory analysis of the downloaded scRNA-seq data, and 41 key genes were identified through weighted correlation network analysis (WGCNA) of the bulk RNA-seq data. Three key genes: Hpgd, Npr3 and Fbln2 were identified by taking intersection of the key genes obtained above, and Hpgd was finally selected for subsequent verification. The human pulmonary artery endothelial cells (hPAECs) were treated with hypoxia for different periods of time, and it was found that the expression of Hpgd decreased in hypoxia-treated hPAECs in a time-dependent manner. In order to further confirm whether Hpgd affects the occurrence and development of HPH, Hpgd was overexpressed in hPAECs.

**Results:**

Hpgd was confirmed to regulate the proliferation activity, apoptosis level, adhesiveness and angiogenesis ability of hypoxia-treated hPAECs through multiple experiments.

**Conclusions:**

Downregulation of Hpgd can improve the proliferation activity, reduce apoptosis, and enhance adhesion and angiogenesis in endothelial cells (ECs), thus promoting the occurrence and development of HPH.

**Supplementary Information:**

The online version contains supplementary material available at 10.1186/s12890-023-02401-y.

## Background

Pulmonary arterial hypertension (PAH) is a progressive occlusive pulmonary vascular disease caused by proliferation of endothelial cells (ECs) and smooth muscle cells (SMCs) as well as the occlusion of small peripheral pulmonary arteries induced by in situ thrombosis. There are multiple causes and a high mortality of PAH [1]. According to the classification of the World Health Organization (WHO), PAH can be divided into 5 subtypes, and hypoxic pulmonary hypertension (HPH), as one of the most common types, belongs to the third subtype [2]. HPH is commonly seen in chronic hypoxic lung diseases, including chronic obstructive pulmonary disease (COPD), obstructive sleep apnea syndrome, interstitial lung disease and chronic mountain sickness [3]. It is caused by hypoxia-induced endothelium injury, imbalance of vascular endothelial synthesis and secretion of various vasodilators, leading to early pulmonary vasoconstriction and pulmonary vascular remodeling. Therefore, it is believed that hypoxic pulmonary vascular remodeling is an important pathological basis of HPH [4]. The development of HPH is accompanied by inflammatory responses and oxidative stress [5], and is related to the worsening of symptoms and poor prognosis caused by elevated arterial pressure and right ventricular hypertrophy [6], but the exact pathogenesis of HPH is still unclear. At present, it has been clinically shown that long-term oxygen therapy can effectively prolong the survival of patients with COPD, but it can only slightly reduce the pulmonary artery pressure of patients [7]. Targeted drugs such as pulmonary vasodilators have not been shown to be effective in the clinical treatment of HPH [8]. Therefore, it is necessary to further explore the exact pathogenesis of HPH to provide more possibilities for the treatment of HPH.

ECs play important roles in the pathogenesis of HPH. Previous studies have demonstrated that up-regulation of HIF-1 and VEGF expression in ECs can induce vascular remodeling in HPH [9]. ECs and SMCs mediate vascular remodeling and PAH through FoxM1 signaling interactions [10]. The up-regulation of endothelial DKK1 (Dickkopf 1) can promote the development of PAH through the Sp1/SHMT2 pathway [11]. The heterogeneity of vascular ECs [12] may be a key factor for the ineffective treatment of HPH with current drugs [13], but fortunately, single cell sequencing (scRNA-seq) can well solve the problem of cell heterogeneity, so as to reveal the complex and rare cell population, the regulatory relationship between genes, and track the trajectories of different cell lineages during development. At present, a large number of studies were found to combinedly make use of scRNA-seq and bulk RNA-seq data for bioinformatics analysis [14, 15], and the results obtained complement each other, making the research more detailed and in-depth.

In this study, the scRNA-seq and bulk RNA-seq data were downloaded from the public database of Gene Expression Omnibus (GEO) (http://www.ncbi.nlm.nih.gov/geo) for joint analysis. Cell subpopulation identification and trajectory analysis were carried out on the scRNA-seq data, and differentially expressed genes (DEGs) analysis and WGCNA were conducted on the bulk RNA-seq data to find out the DEGs highly correlated with HPH. Three key genes: Hpgd, Npr3 and Fbln2 were obtained by taking intersection of the important genes obtained from the two datasets, and Hpgd was selected for subsequent verification. Hpgd was verified by Western blot and qRT-PCR, and was confirmed to be significantly down-regulated in hypoxia-treated human pulmonary artery endothelial cells (hPAECs). To further figure out the role of Hpgd in the pathogenesis of HPH, we overexpressed Hpgd in ECs in vitro, and measured ECs’ proliferative activity, apoptosis level, adhesion and angiogenesis. In a word, through bioinformatics analysis and experimental validation, this study is expected to provide theoretical support for elucidating the pathogenesis of HPH and discover potential therapeutic target genes of HPH.

## Materials and methods

### Data retrieval

GEO is a public functional genomics data repository. GSE154959 [16] and GSE131425 were screened out from GEO. GSE154959 is a dataset containing the scRNA-seq data of lung ECs isolated from three controls and three PAH mice. GSE131425 contains bulk RNA-seq data of lung tissues from six controls and four hypoxia-induced PAH mice.

### scRNA-seq data processing

The scRNA-seq data were analyzed using the “Seurat” package (version 4.0.4). Cell quality control was carried out based on the following criteria: (1) genes detected in < 5 cells were excluded; (2) cells with < 300 total detected genes were excluded; and (3) cells with ≥ 20% of mitochondria-expressed genes were excluded. The functions of “IntegrateData” and “NormalizeData” in the “Seurat” package were used for batch effect elimination and normalization between the six samples. Subsequently, the Uniform Manifold Approximation and Projection (UMAP) algorithm was used for dimensionality reduction of the 30 initial PCs, and cluster analysis was performed for all units. The cell clusters were subjected to cell type annotation according to the cell marker genes (Supplementary Table 1) from literature and the CellMarker database (http://xteam.xbio.top/CellMarker/).

### Subpopulation identification of pulmonary ECs [17]

The subpopulation markers of ECs were identified based on previously reported literature [18, 19]. The “FindConservedMarkers” function in “Seurat” package was used to identify proliferating cells’ markers, and we provided the detailed data list of marker genes corresponding to proliferating cells (Supplementary Table 2). The signature of proliferating cells was calculated using the average expression of proliferating cells’ markers in GSE131425.

### Trajectory analysis of arterial and proliferating cells

Trajectory analysis reconstructs the change process of cells over time by constructing the change trajectories between cells. To map the differentiation of cell subsets in ECs in HPH, we use the “plot_cell_trajectory” in “Monocle2” package to perform trajectory analysis. The “FindConservedMarkers” function in “Seurat” package was used to select the top 2,000 highly variable features (HVGs) for construction of trajectories. The “BEAM” function in Monocle2 was used to define branch-specific genes.

### Differential analysis and functional enrichment analysis

Relevant DEGs were screened using the “DESeq2” package. Heatmap of the DEGs was drawn using the “pheatmap” package. Gene Ontology (GO) and Kyoto Encyclopedia of Genes and Genomes (KEGG) enrichment analyses [20–22] were performed using the “clusterProfiler” package. DEGs with padj < 0.05 and |log2FC| > 1 were considered statistically significant.

### Weighted gene co-expression network analysis (WGCNA)

WGCNA is designed to show the co-expression relationships between genes. It is a systems biological approach to describe gene association patterns between different samples. WGCNA can be used to identify gene sets with high covariation, and to identify candidate biomarker genes or therapeutic targets based on the endogeneity of gene sets and the association between gene sets and phenotypes. We calculated the correlation value of gene expression in GSE131425, and carried out suborder operation. The soft threshold was adjusted to 8 (scale free R^2^ = 0.8) to construct a scale-free network. R^2^ = 8 was used to construct an adjacency matrix of the differential genes, which was then transformed into a topological overlap matrix (TOM). Hierarchical clustering was then performed for module identification. Finally, the feature genes were calculated, the modules were hierarchically clustered, and similar modules were merged. 7 modules were subsequently identified.

### Cell model preparation and grouping

An in vitro cell model was built by treating hPAECs with hypoxia to simulate HPH. The over-expression vector of Hpgd (OE-Hpgd) was constructed and transfected into hPAECs. At the same time, the empty plasmid (OE-NC) was transferred into hPAECs as a control. The transfected hPAECs were treated with hypoxia for 24 h, and were divided into hypoxia + OE-Hpgd group and hypoxia + OE-NC group.

### Cell culture

The hPAECs (#CP-H002) used in this study were purchased from Pricella, inoculated in the complete culture medium for hPAECs (Pricella, #CM-H002), and cultured in an incubator with 5% CO_2_ at 37℃.

### Western blot

The total protein was extracted from the cell lysate containing 1% protease inhibitor, and the protein in the samples was quantified by BCA method, electrophoresis, membrane transfer, and antibody incubation. The primary antibodies were Hpgd (Abcam, #ab187160), Bax (Abcam, #ab32503) and Bcl-2 (Abcam, #ab32124). GAPDH (Abcam, #ab8245) was selected as the internal reference gene. The optimum concentration was 0.418 µg/ml for Hpgd, 0.11 µg/ml for Bax, 0.4 µg/ml for Bcl-2, and 2 µg/ml for GAPDH. The protein strips were exposed and analyzed using Image J software.

### qRT-PCR

Total RNA was extracted using the TRIzol kit (ThermoFisher, USA). The cDNA was obtained using the PrimeScript RT kit (TaKaRa, Japan), and then the cDNA template was diluted with RNase-free water. qPCR was performed according to the instructions of SYBR Green PCR kit (ThermoFisher, USA). The total reaction system was 10 µl. The condition of qRT-PCR: pre-denaturation at 95℃ for 15 min, denaturation at 94℃ for 15s, annealing at 55℃ for 30s, extension at 72℃ for 30s, and a total of 40 cycles. The primer sequences are as follows: Hpgd-forward primer (F): GCAAGCGAACCATTCCACTCTTTG; Hpgd-reverse primer (R): ACAGTTTCACATTCCCACCCATCAG; GAPDH-F: GATCATCAGCAATGCCTCCT; GAPDH-R: TGTGGTCATGAGTCCTTCCA. The qRT-PCR (#A28139) instrument was purchased from Thermo Fisher.

### Assessment of the proliferation and apoptosis of hPAECs

According to the manufacturer’s instructions, the levels of proliferation and apoptosis of hPAECs were evaluated using Bromodeoxyuridine (BrdU) incorporation assay (spbio, China) and in situ Brdu-red DNA fragmentation (TUNEL) apoptosis assay kit (Beyotime, China), and the degree of apoptosis was evaluated by Bax/Bcl-2 ratio.

### Adhesion and angiogenesis assay of the ECs

Adhesion experiments of ECs were performed according to the instructions of the cell adhesion kit (AAT Bioquest, 23,010). Additionally, the pre-cooled Matrigel matrix glue was added into the 24-well plates, and 500 µl of the cell suspension was added into each well. The results of angiogenesis assay were observed after routine culture in the cell incubator for 2-6 h. Image J and NIS-Elements BR Analysis (Nikon) were used to quantify adherent cells and tube formation.

### Statistical analysis

The experimental data were processed using R language and GraphPad Prism 8.0. *P* > 0.05 denotes statistically significant difference.

## Results

### Differential analysis

**Fig. 1:**
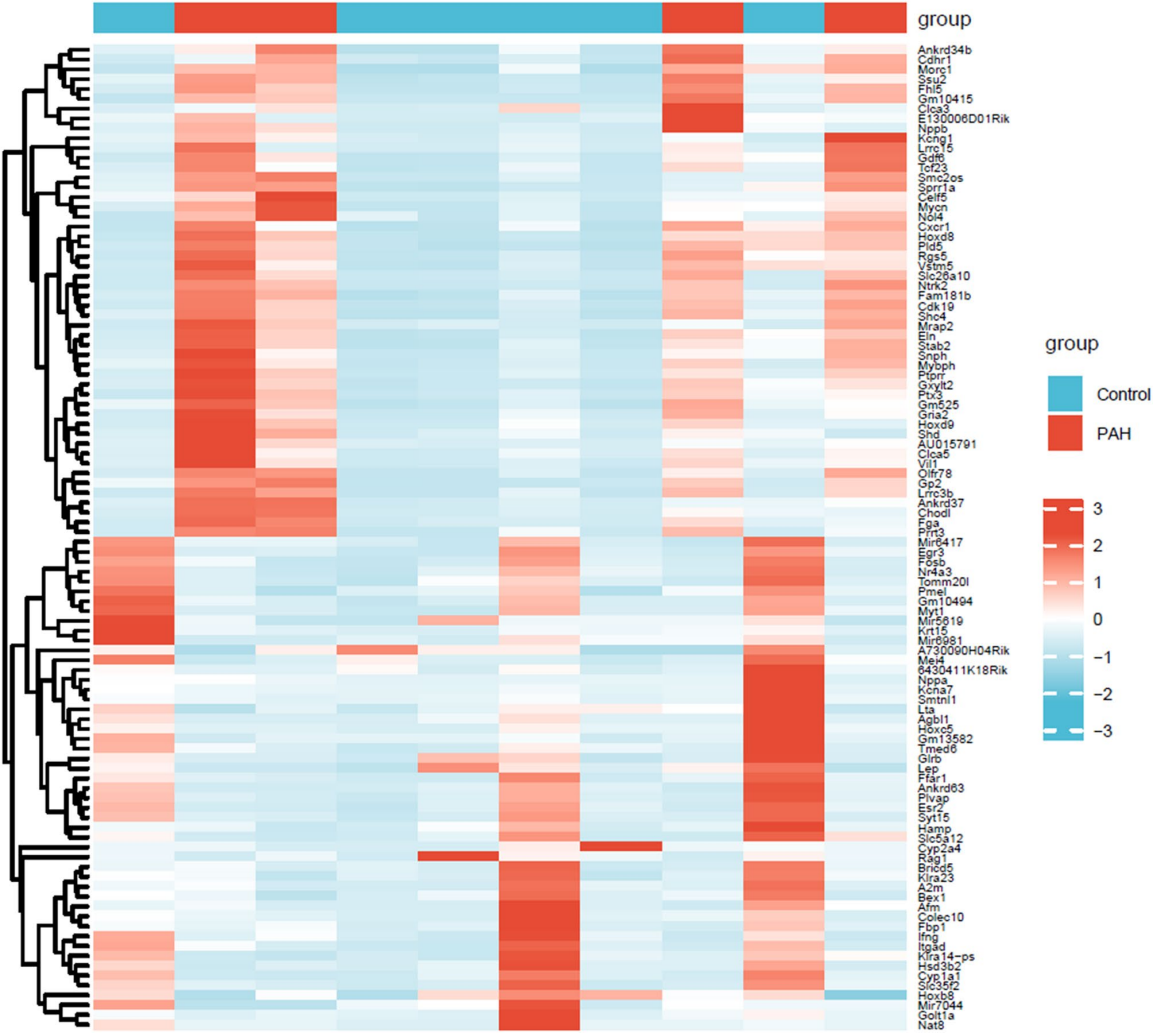
Heatmap of the first 100 DEGs.

The GSE131425 dataset were subjected to DEGs analysis. Based on *P* < 0.05 and |log_2_FC| > 1, 454 DEGs were identified, of which 277 were up-regulated and 277 were down-regulated (Fig. 1& Supplementary Table 3).

### Heterogeneity of the ECs

**Fig. 2:**
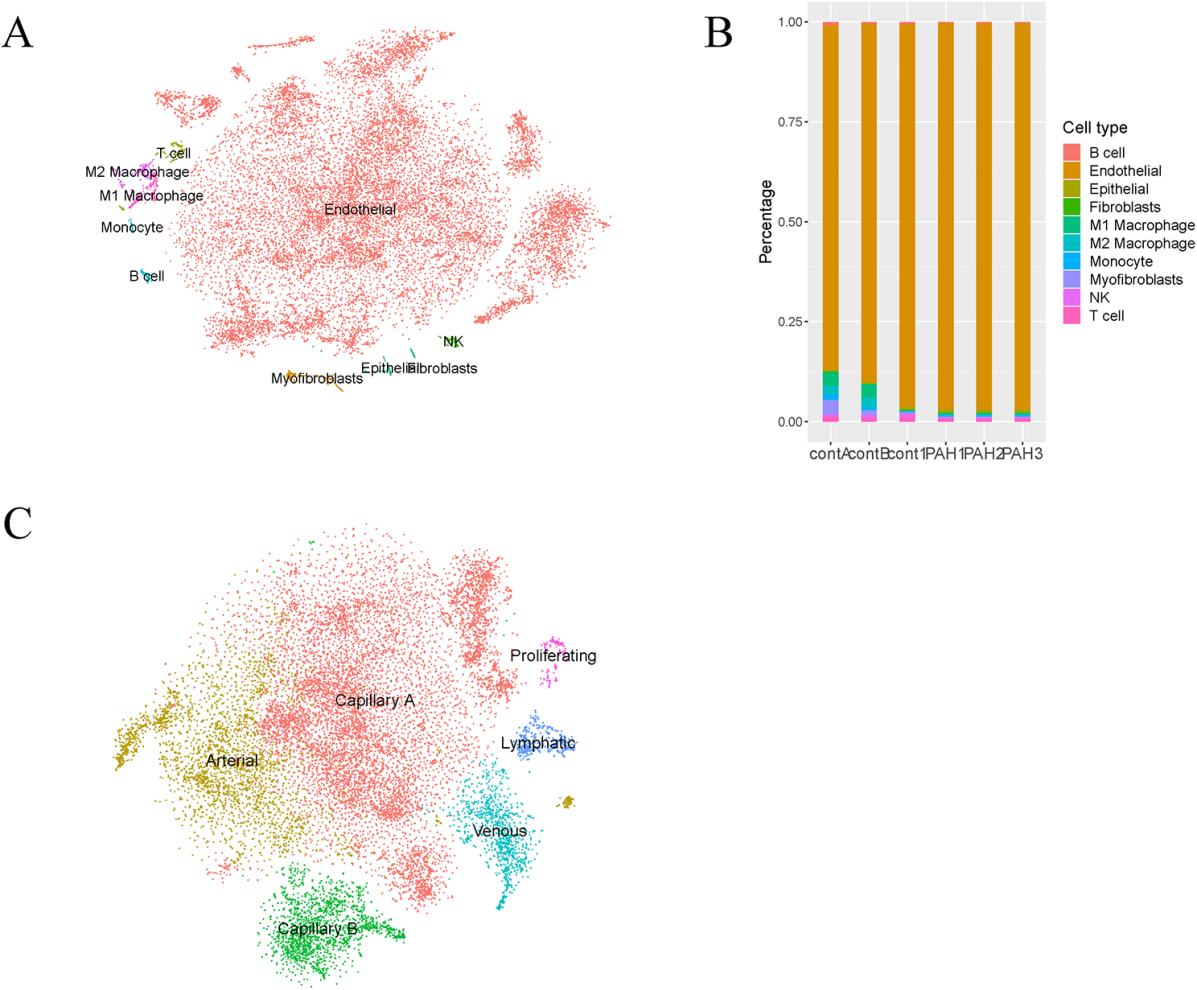
Heterogeneity of the ECs. **(A)** The UMAP of cell types. **(B)** The proportion of different cell types in the samples. **(C)** Subpopulation distribution of the ECs.

The Seurat object contained 28,940 cells and 16,980 genes after quality control and batch correction. These cells were clustered into 21 clusters. According to the expression of cell marker genes in clusters obtained from literature review and CellMarker database, cell type annotations were made for the clusters (Fig. 2A). The ECs accounted for the largest proportion of all the samples (Fig. 2B). 6 subpopulations of the ECs were revealed through further analysis, and they were capillary A, capillary B, venous, arterial, lymphatic and proliferating cells (Fig. 2C). 183 proliferating cell markers were identified with the aid of “FindConservedMarkers” function.

### WGCNA

**Fig. 3:**
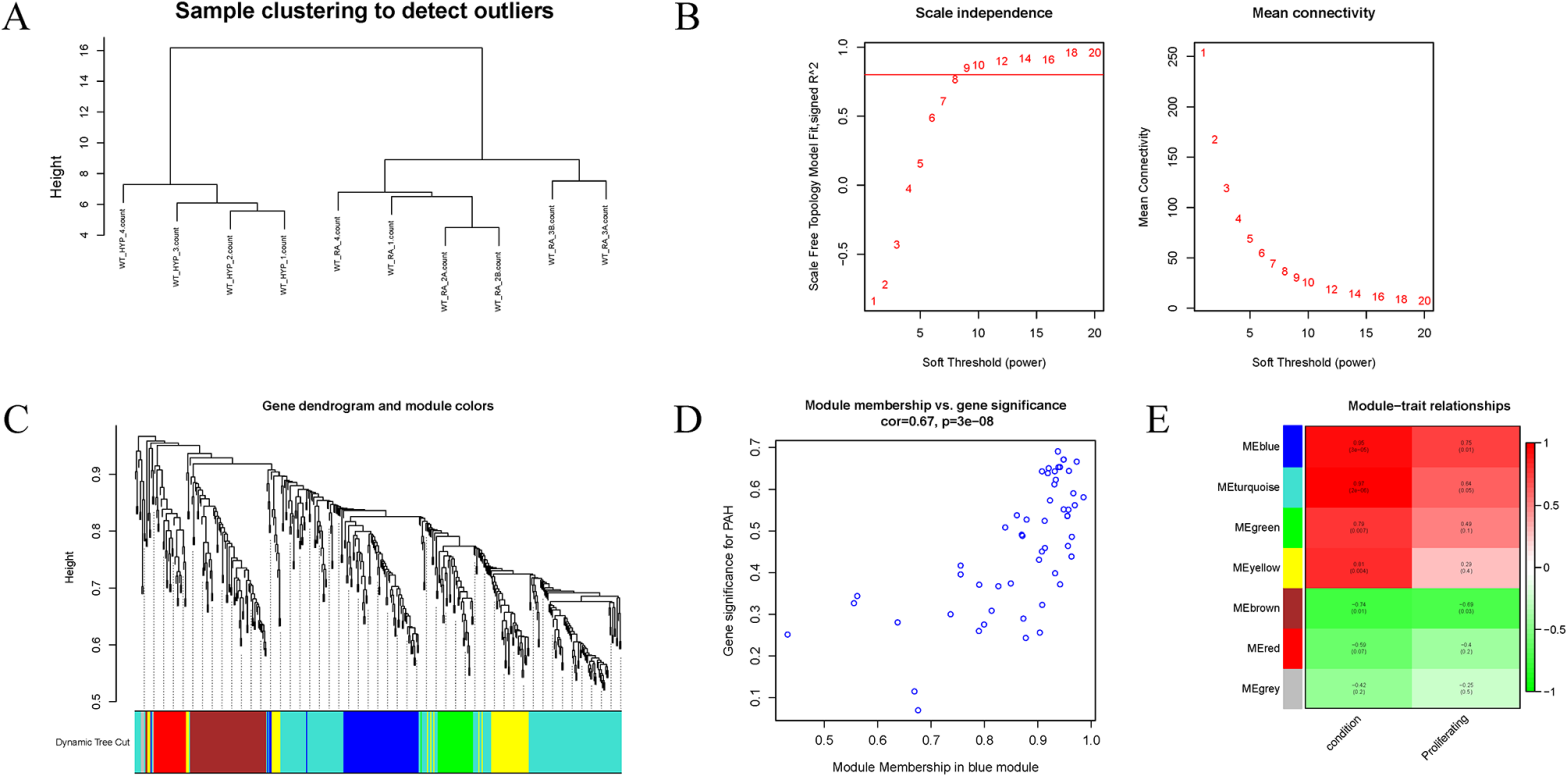
WGCNA. **(A)** Clustering dendrogram of 10 samples. **(B)** Determination of the soft-threshold power. **(C)** Gene dendrogram and module colors. **(D)** A scatterplot of GS for PAH vs. MM in the blue module. **(E)** Correlation analysis between each module and indicator

**Fig. 4:**
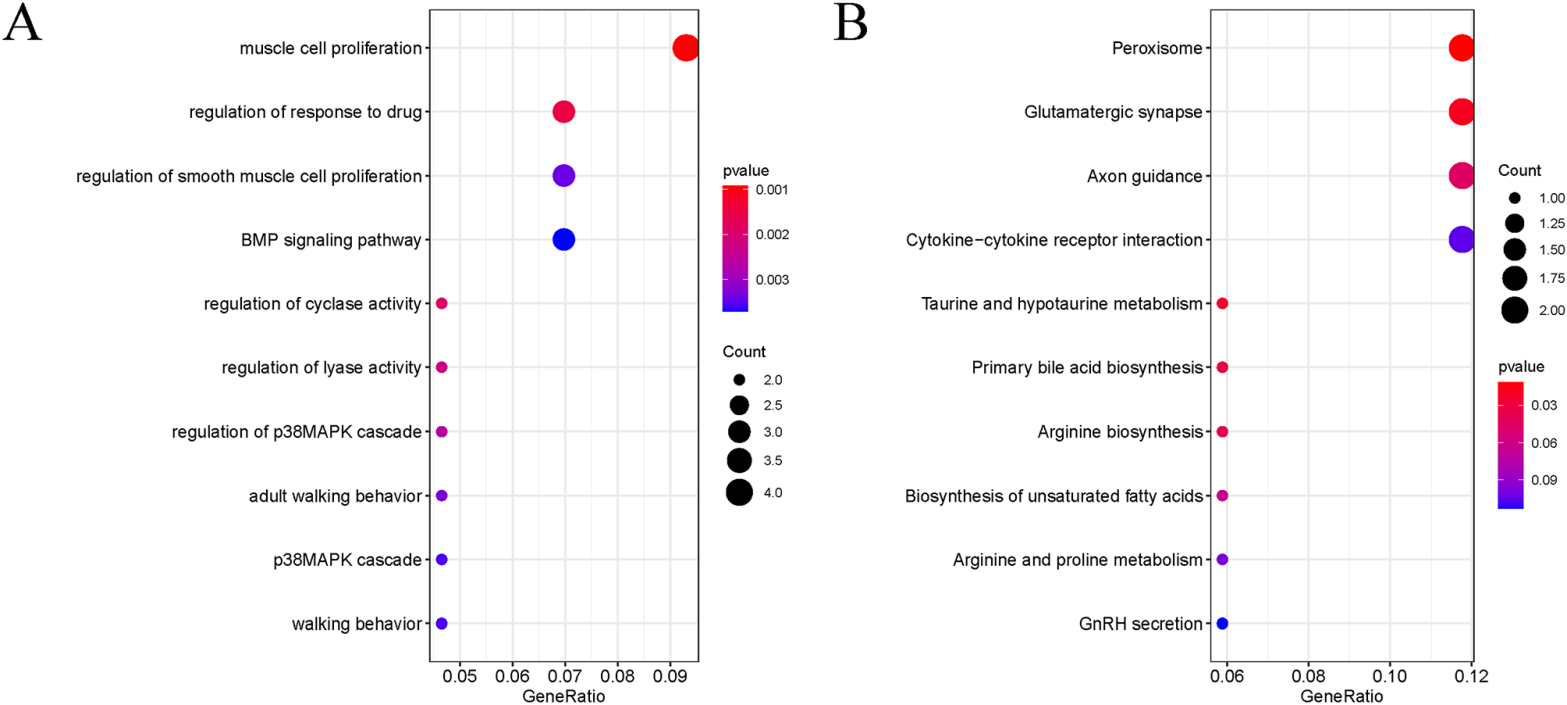
Functional enrichment analysis of the key genes in the blue module. **(A)** GO enrichment analysis. **(B)** KEGG pathway enrichment analysis

Pearson’s correlation coefficient was used to cluster the samples, and a sample clustering tree was drawn correspondingly (Fig. 3A). The soft threshold was adjusted to 8 (R^2^ = 0.8) for scale-free network construction (Fig. 3B). Next, the adjacency matrix was built and the TOM was constructed. Finally, 7 modules were identified based on average hierarchical clustering and dynamic tree clipping (Fig. 3C). The correlation analysis of different modules and indicators (HPH and the proliferating cell signature) showed that the blue module was highly correlated with HPH and the proliferating cell signature (Fig. 3D, E). Therefore, it was chosen as the clinically important module for follow-up analysis. The blue module contained 181 genes, and 41 key genes were screened out according to Gene Significance (GS) > 0.9 and Module Membership (MM) > 0.9. GO and KEGG enrichment analysis was performed on the key genes (Fig. 4A, B). Most of these genes were shown to be enriched in muscle cell proliferation, peroxisome, cytokine-cytokine receptor interaction, and other functions.

### Trajectory analysis

**Fig. 5:**
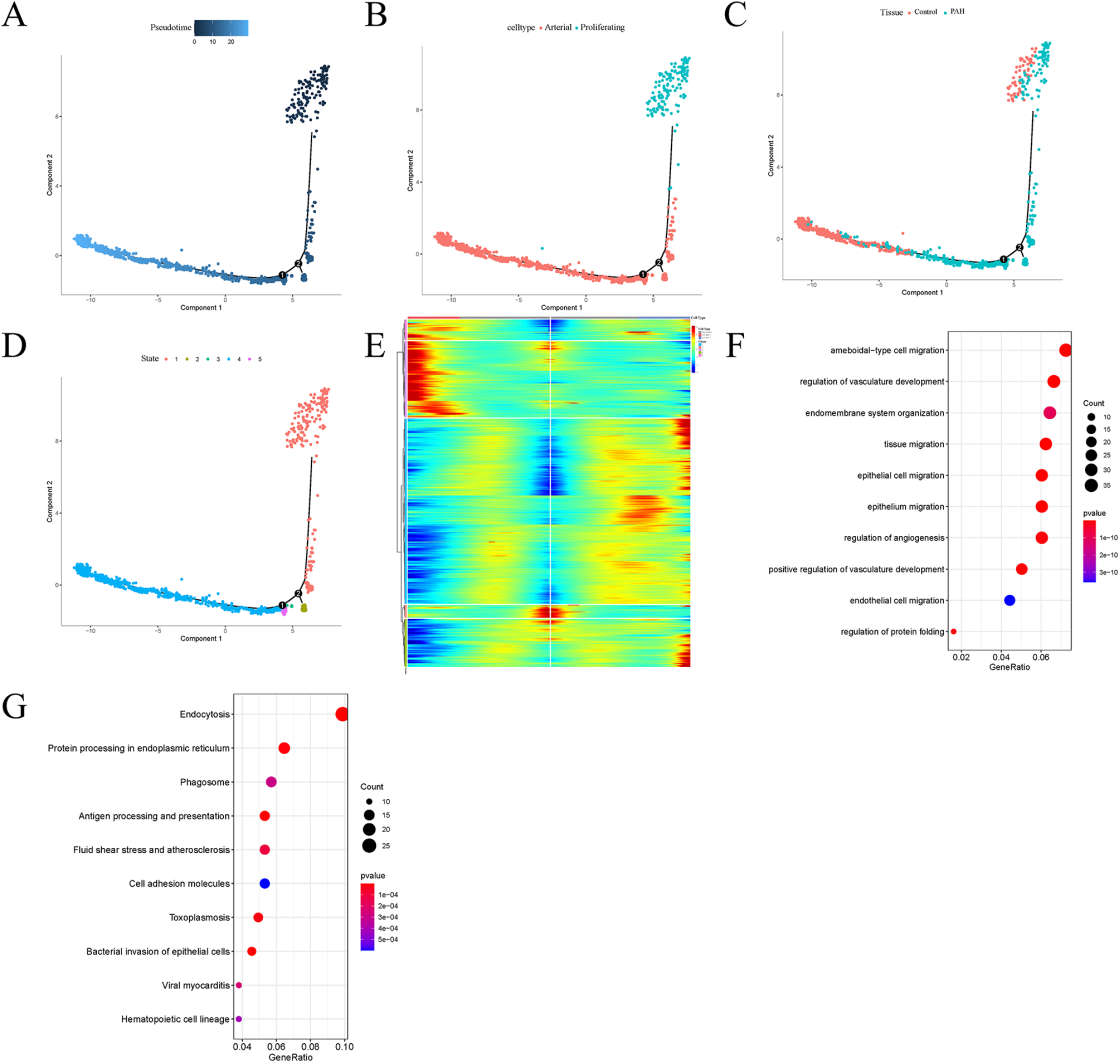
Trajectory analysis and functional enrichment analysis. Trajectories plotted by **(A)** pseudo-time, **(B)** cell type, **(C)** organization and **(D)** state. **(E) **Heatmap of branch-dependent genes. **(F)** GO enrichment analysis of the genes in cluster 1. **(G)** KEGG pathway enrichment analysis of the genes in cluster 1

The trajectory analysis projects all arterial cells and proliferating cells onto five states. The results showed that the cells ranged from proliferating cells to arterial cells in the pseudo-time line, and the proliferating cells were mostly from the HPH group (Fig. 5A-C). According to cell differentiation, cell was divided into five states (Fig. 5D). Branch point 1 was analyzed using BEAM method and the results were clustered (threshold, qval < 1e-6). These genes can be clustered into 5 categories (Fig. 5E), where pre-branch represents state 3, cell fate 1 represents state 4, and cell fate 2 represents state 5. Cluster 1 contains a large number of genes, and the overall trend is roughly consistent, so we choose cluster 1 for subsequent analysis. Cluster 1 contains 523 genes. GO and KEGG enrichment analysis were performed on the genes in cluster 1 (Fig. 5F, G), and the results showed that most of the genes were enriched in the regulation of vasculature development, endocytosis, phagosome and other related functions.

### Hub genes

**Fig. 6:**
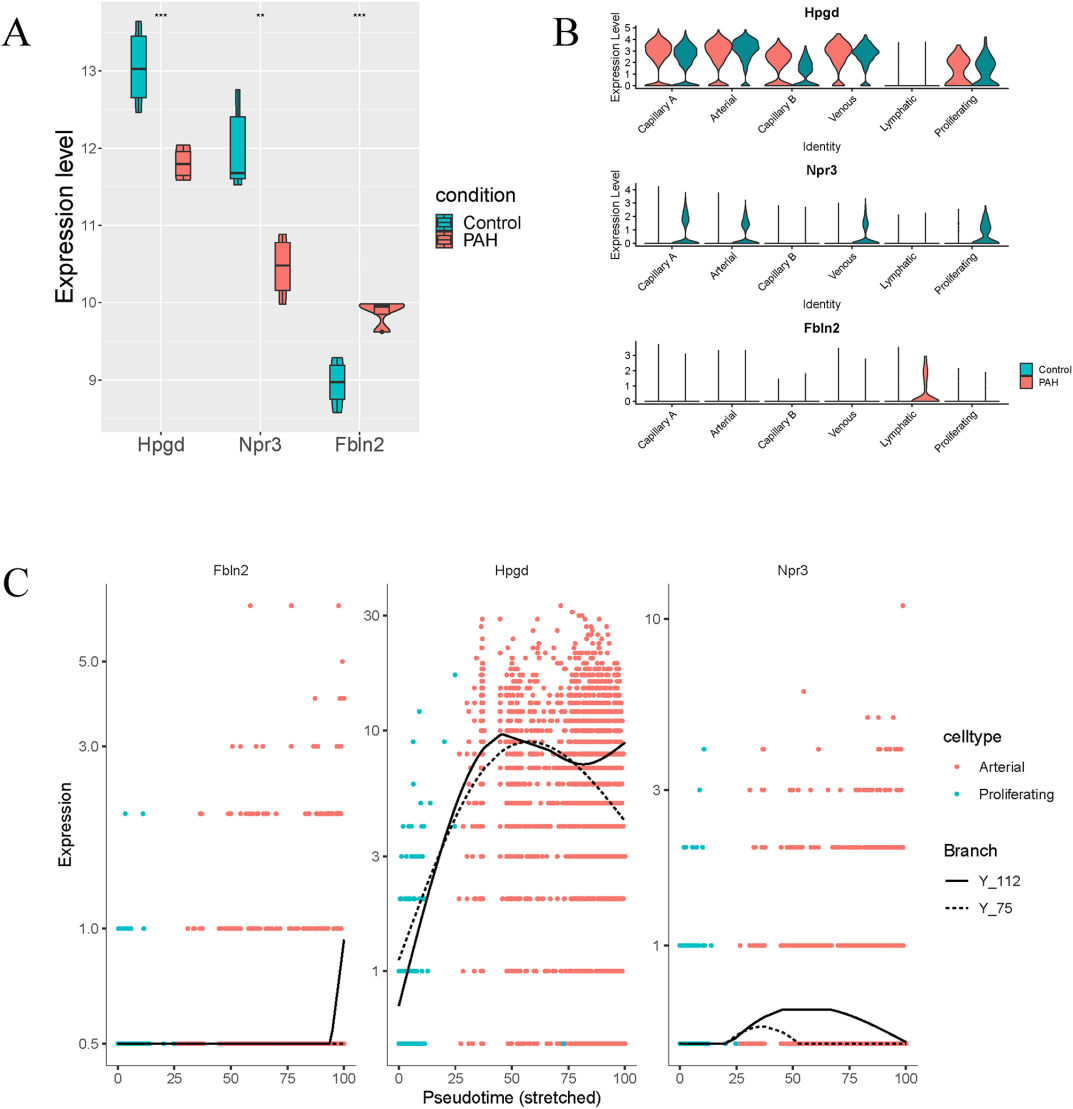
The expression of Hpgd, Npr3 and Fbln2. Gene expression in **(A)** bulk RNA-seq data, **(B)** scRNA-seq data and **(C)** trajectory analysis

Three key genes (Hpgd, Npr3 and Fbln2) were finally screened out through intersection between the key genes of the blue module and the genes in cluster 1. Compared with the control group, Hpgd and Npr3 were down-regulated, and Fbln2 was up-regulated in PAH (Fig. 6A). The expression level of Hpgd in ECs was much higher than that of Npr3 and Fbln2 (Fig. 6B). In addition, through trajectory analysis, Hpgd showed an obvious trend of change in the process of cell differentiation (Fig. 6C), so Hpgd was selected for cell experiment verification.

### Hpgd expression decreased in hypoxia-treated hPAECs

**Fig. 7:**
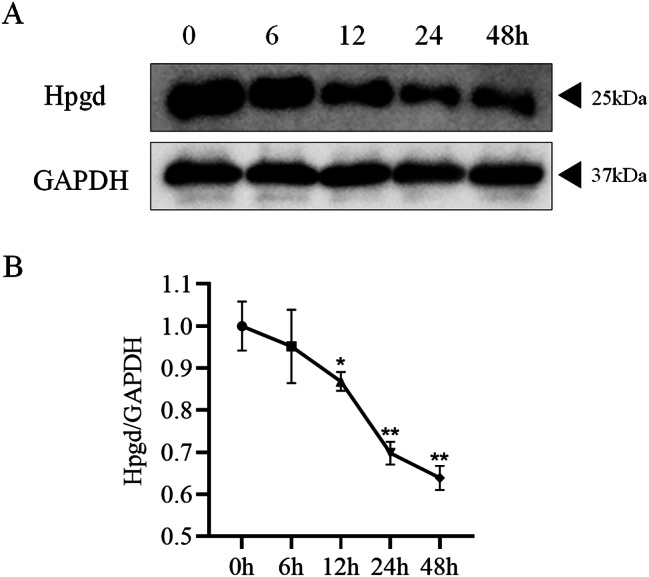
The expression levels of Hpgd in hPAECs treated with hypoxia for 0, 6, 12, 24 and 48 h. **(A)** Western Blot results showed that the expression of Hpgd decreased in hypoxia-treated hPAECs. **(B)** qRT-PCR showed that the longer the time of hypoxic treatment of hPAECs, the lower the expression of Hpgd. (^*^P<0.05, ^**^P<0.01)

hPAECs were treated with different durations of hypoxia (0, 6, 12, 24, 48 h) in order to simulate HPH cell model in vitro. Western blot and qRT-PCR were used to detect the expression of Hpgd in hPAECs treated with different durations of hypoxia. Hpgd expression decreased in a time-dependent manner in hypoxia-treated hPAECs (Fig. 7A, B). This result was consistent with the results of bioinformatics analysis above.

### Effects of Hpgd on the proliferation and apoptosis of hypoxia-treated hPAECs

**Fig. 8:**
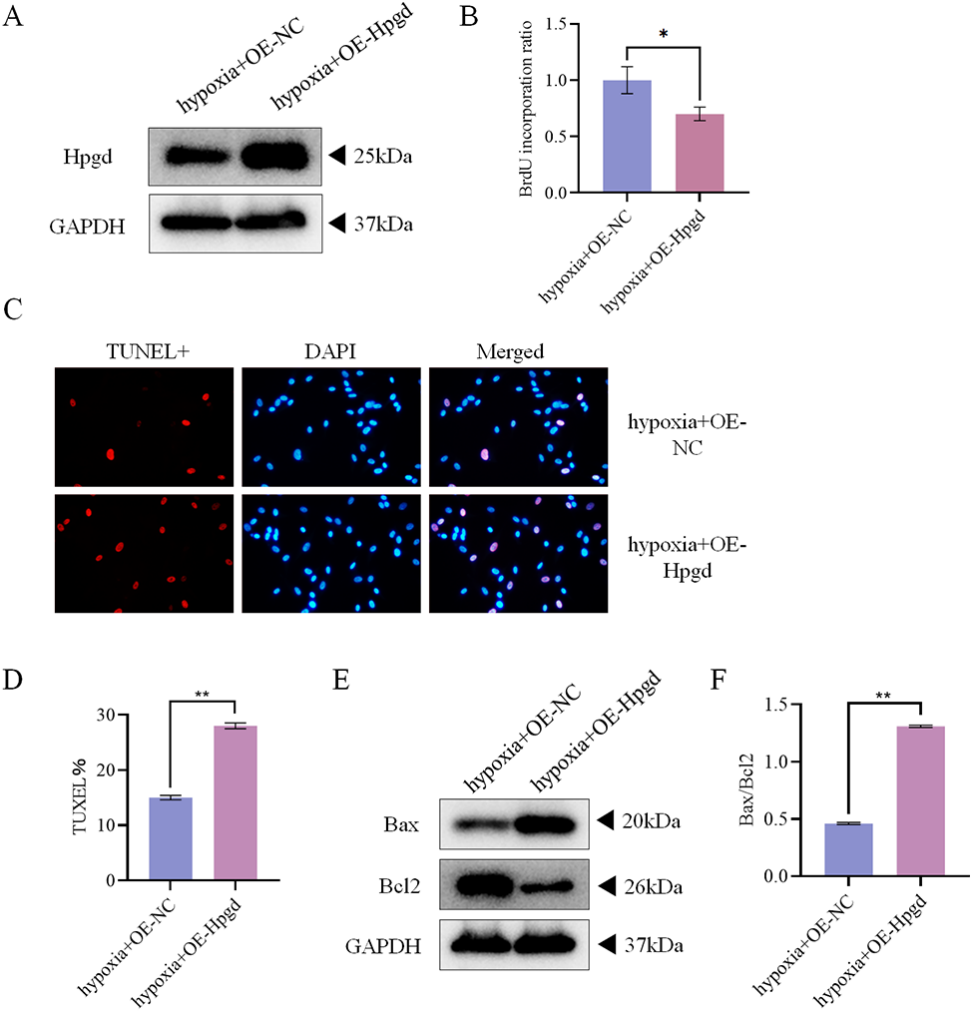
Effects of Hpgd on the proliferation and apoptosis of hPAECs. **(A)** Western blot verified that hPAECs was successfully transfected with OE-Hpgd. **(B)** BrdU incorporation assay showed that OE-Hpgd inhibited cell proliferation. **(C)** TUNEL staining results. **(D)** TUNEL staining showed that OE-Hpgd increased the number of apoptotic cells. **(E)** The expression of apoptosis-related proteins shown by Western blot. **(F)** The ratio of Bax/Bcl-2 expression showed that the degree of apoptosis was deepened. (^*^P<0.05, ^**^P<0.01)

It is known that the expression of Hpgd decreased in hypoxia-treated hPAECs. In order to study the effect of Hpgd on the proliferation and apoptosis of hPAECs, OE-NC/OE-Hpgd was transfected into hPAECs (Fig. 8A). The proliferation activity of hPAECs was detected by BrdU incorporation assay, and the apoptosis was detected by TUNEL staining. It was found that compared with the hypoxia + OE-NC group, the proliferation activity in the hypoxia + OE-Hpgd group decreased, and the number of apoptotic cells increased (Fig. 8B-D). Western blot was used to detect the expression of apoptosis-related proteins (Bax and Bcl2). Compared with the hypoxia + OE-NC group, the Bax/Bcl2 ratio in the hypoxia + OE-Hpgd group increased (Fig. 8E, F). These results indicated that Hpgd regulated the proliferative activity and apoptosis of hypoxia-treated hPAECs.

### Effects of Hpgd on the adhesion and angiogenesis of hypoxia-treated hPAECs

**Fig. 9:**
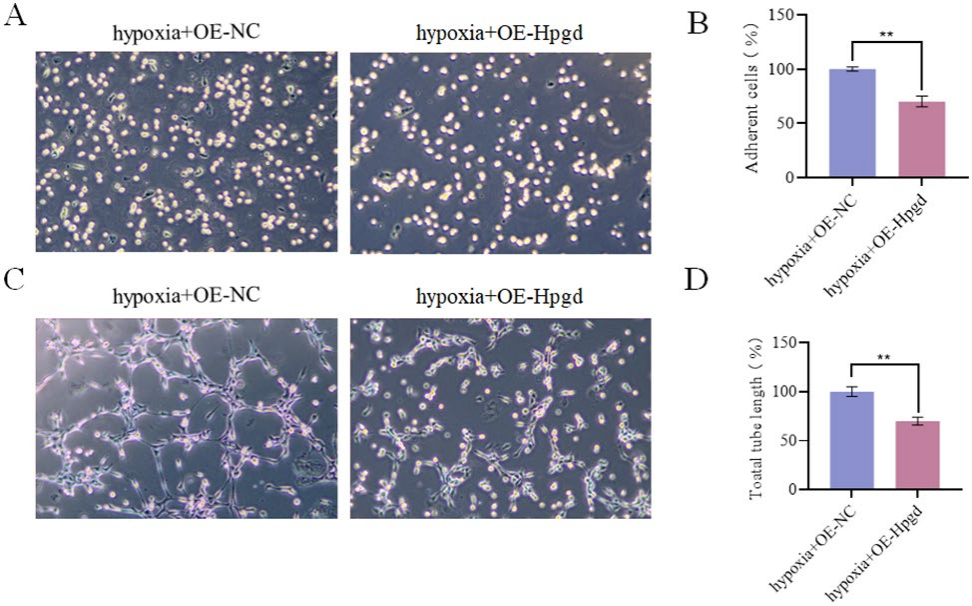
Effects of Hpgd on the adhesion and angiogenesis of hPAECs. **(A)** The microscope results showed that OE-Hpgd reduced the adhesion of hPAECs. **(B)** Statistical histogram of the adherent cells. **(C)** The microscope results showed that OE-Hpgd reduced the angiogenesis capability. **(D)** Statistical bar chart of the number of tubules. (^**^P<0.01)

In order to investigate the effect of Hpgd on the adhesion and angiogenesis of hPAECs, the levels of adhesion and angiogenesis of hPAECs transfected with OE-NC/OE-Hpgd were tested. It was found that both adherent cells and angiogenesis (number of tubules) were reduced in the hypoxia + OE-Hpgd group when compared with the hypoxia + OE-NC group (Fig. 9A-D), indicating that Hpgd regulated the adhesion and angiogenesis of hypoxia-treated hPAECs.

## Discussion

HPH is caused by hypoxia-induced pulmonary vasoconstriction, thus increasing pulmonary artery pressure. At present, due to the limited detection methods, HPH is less liable to be detected in time. Moreover, HPH is mostly induced by basic pulmonary diseases that complicate the course of chronic diseases [23]. Current treatments for HPH generally give priority to the underlying lung diseases [24, 25]. However, the pathogenesis of HPH remains unclear.

In order to investigate the mechanism of HPH, we downloaded the scRNA-seq dataset GSE154959 and the bulk RNA-seq dataset GSE131425 from GEO. According to the identification of cell types by using the scRNA-seq data, it was found that ECs accounted for the largest proportion and had six subtypes: capillary A, capillary B, venous, arterial, lymphatic, proliferating cells that suggested the heterogeneity of ECs. Recent scRNA-seq profiles of mouse ECs show that ECs are heterogeneous in the brain, muscles, heart, kidney and other tissues [26, 27]. The heterogeneity of ECs has been established early in the embryonic development, and is gradually adjusted and enhanced with each developmental stage. At the same time, such heterogeneity may also change under different pathological conditions [28]. According to existing studies, ECs proliferate abnormally with the occurrence of HPH, causing vascular remodeling [29]. Through trajectory analysis of arterial cells and proliferating cells, 523 key genes were identified, and they were revealed to be enriched in the regulation of vasculature development, endocytosis, phagosome by GO and KEGG analysis. To some extent, key genes associated with the transformation from proliferating cells to arterial cells were considered relevant to vascular remodeling. Piperlongumine has been shown to treat HPH by regulating autophagy and reducing vascular remodeling [30]. Fasudil is known to interfere with vascular remodeling, reduce proliferation and apoptosis of pulmonary ECs, and alleviate the occurrence of HPH [31]. Galectin-3, expressed in vascular SMCs, promotes PAH by regulating changes in the proliferation, apoptosis, and fibrosis of the cells [32]. Nicotinic acid can inhibit vascular remodeling by releasing H-PGDS from macrophages and promoting the production of PGD2 in lung tissues, thus improving the progression of PAH [33]. T cells are regulated to up-regulate pulmonary and right ventricular vascular protective protein COX-2 (cyclooxygenase 2) in ECs to counteract the pulmonary vascular damage produced in HPH [34].

WGCNA of the bulk RNA-seq data was performed to identify important clinical modules, and 41 key genes were screened out based on GS > 0.9 and MM > 0.9. Most of these genes were shown by GO and KEGG enrichment analysis to be enriched in the regulation of vascular development, SMC proliferation and immunity-related functions. 3 key genes (Hpgd, Npr3 and Fbln2) were obtained through intersection of genes screened from the two datasets mentioned above, and their expressions were analyzed in the meantime. Compared with the control group, Hpgd and Npr3 were down-regulated in PAH, and Fbln2 was up-regulated in PAH.

Natriuretic peptide receptor 3 (Npr3) encodes a receptor clearance gene for final degradation by binding and internalizing natriuretic peptides (NPs). It protects cardiomyocytes from apoptosis through inhibition of cytosolic BRCA1 and TNF-α, which are regulators of apoptosis [35]. Fibulin-2 (Fbln2) codes for a gene that secretes an extracellular matrix glycoprotein and is involved in tissue development and remodeling. Fbln2 is expressed in the epithelial-mesenchymal transition of the central endometrial cushion matrix during embryonic heart development [36]. According to Shaukat’s study, the expression of Fbln2 is a key regulator of angiotensin II-induced TGF-β signaling and subsequent myocardial fibrosis [37]. Hpgd (15-hydroxyprostaglandin dehydrogenase) encodes a member of the short chain nonmetallic enzyme alcohol dehydrogenase protein family, and can activate STAT3 and AKT pathways to promote proliferation, migration and anchorage-independent growth of cervical cancer cells [38]. Deletion of Hpgd in regulatory T cells (Treg cells) in mice leads to abnormal proliferation and accumulation of functionally impaired Treg cells, inducing local inflammation [39]. miR-106b-5p regulates the progression of esophageal squamous cell carcinoma by binding to Hpgd [40]. However, there has been no corresponding study on Hpgd in HPH. Therefore, we verified the role of Hpgd by in vitro experiments. After hypoxic treatment of hPAECs, the expression of Hpgd was decreased in a time-dependent pattern, which was consistent with the results drawn from bioinformatics analysis. Hpgd overexpression was revealed to regulate the proliferative activity, apoptosis level, adhesion and angiogenesis capability of hypoxia-treated hPAECs.

## Conclusion

In this study, bioinformatics analysis was carried out based on both of the scRNA-seq data and bulk RNA-seq data, through which we found that Hpgd may play a key role in the occurrence and development of HPH. Through cellular experimental verification, Hpgd was shown to decrease the proliferation capacity, increase the level of apoptosis, and reduce the adhesion and angiogenesis ability of ECs. This study deepens the current understanding of the pathogenesis of HPH, which may enable us to conduct intervention therapy for HPH targeting Hpgd.

## Electronic supplementary material

Below is the link to the electronic supplementary material.

Supplementary Material 1

Supplementary Material 2

Supplementary Material 3

Supplementary Material 4

Supplementary Material 5

Supplementary Material 6

Supplementary Material 7

Supplementary Material 8

## Data Availability

The datasets used and/or analysed during the current study are available from the GEO database (http://www.ncbi.nlm.nih.gov/geo) [Accession number: GSE154959 (https://www.ncbi.nlm.nih.gov/geo/query/acc.cgi?acc=GSE154959) & Accession number: GSE131425 (https://www.ncbi.nlm.nih.gov/geo/query/acc.cgi?acc=GSE131425)].

## Abbreviations

HPH: Hypoxic pulmonary hypertension
scRNA-seq: single cell sequencing
RNA-seq: RNA sequencing
GEO: Gene Expression Omnibus
WGCNA: weighted correlation network analysis
hPAECs: human pulmonary artery endothelial cells
ECs: endothelial cells
SMCs: smooth muscle cells
COPD: chronic obstructive pulmonary disease
PAH: Pulmonary arterial hypertension
WHO: World Health Organization
DEGs: differentially expressed genes
UMAP: Uniform Manifold Approximation and Projection
HVGs: highly variable features
GO: Gene Ontology
KEGG: Kyoto Encyclopedia of Genes and Genomes
TOM: topological overlap matrix
BrdU: Bromodeoxyuridine
GS: Gene Significance
MM: Module Membership
Npr3: Natriuretic peptide receptor 3
Fbln2: Fibulin-2
