# Supplementary material for: Hpgd affects the progression of hypoxic pulmonary hypertension by regulating vascular remodeling
Source: BMC Pulm Med. 2023 Apr 13;23:116. doi: 10.1186/s12890-023-02401-y (PMC10103477; doi:10.1186/s12890-023-02401-y)
Supplement: Supplementary file 1 — Supplementary Material 1 [file 12890_2023_2401_MOESM1_ESM.docx]

Supplementary table 1：Cell type annotation of the cell clusters

| #Cancerstemcell | #Basal | #Mast | #Luminalcell | #Endothelialcell | #Epithelialcell | #Smoothmusclecell | #Fibroblasts | #Myofibroblasts | #Monocyte | #Macrophage | #NK | #Tcell | #Bcell |
| --- | --- | --- | --- | --- | --- | --- | --- | --- | --- | --- | --- | --- | --- |
| PROM1 | KRT19 | MS4A2 | AR | PECAM1 | SFN | ACTA1 | MYL9 | MYH11 | LYZ | MARCO | GZMB | CD2 | IGHG1 |
| CD44 | KRT18 | TPSAB1 | KRIT1 | VWF | EPCAM | MCAM | ACTA2 | GJA4 | FCGR3A | CD68 | NKG7 | CD3D | IGKC |
| ABCG2 | KRT14 | TPSB2 | KRT8 | ENG | CDH1 | PDGFRB | DCN | RGS5 | CSF1R | MSR1 | GNLY | CD3E | CD79A |
| BMI1 | KRT5 |  | SLC45A3 |  | CTNNB1 | TAGLN | TNFAIP6 | MT1A | CD163 | FCGR2A |  | PTPRC | MS4A1 |
| ITGB1 | TP63 |  | CP |  |  |  | FBLN1 |  | CD14 |  |  |  |  |
| KIT |  |  | B4GALT1 |  |  |  |  |  |  |  |  |  |  |
