# Supplementary figures and images for: Hpgd affects the progression of hypoxic pulmonary hypertension by regulating vascular remodeling
Source: BMC Pulm Med. 2023 Apr 13;23:116. doi: 10.1186/s12890-023-02401-y (PMC10103477; doi:10.1186/s12890-023-02401-y)

— — — — —

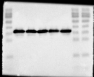

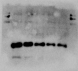

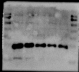

Supplement: Supplementary file 4 — Supplementary Material 4 [file 12890_2023_2401_MOESM4_ESM.pdf]

—



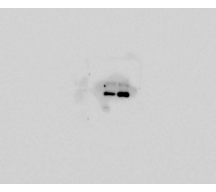

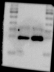

Supplement: Supplementary file 5 — Supplementary Material 5 [file 12890_2023_2401_MOESM5_ESM.pdf]

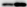

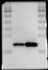

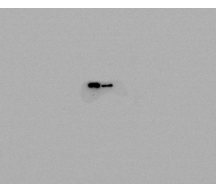

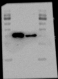

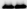

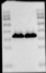

Supplement: Supplementary file 7 — Supplementary Material 7 [file 12890_2023_2401_MOESM7_ESM.pdf]

Fig 9A hypoxia+OE-NC

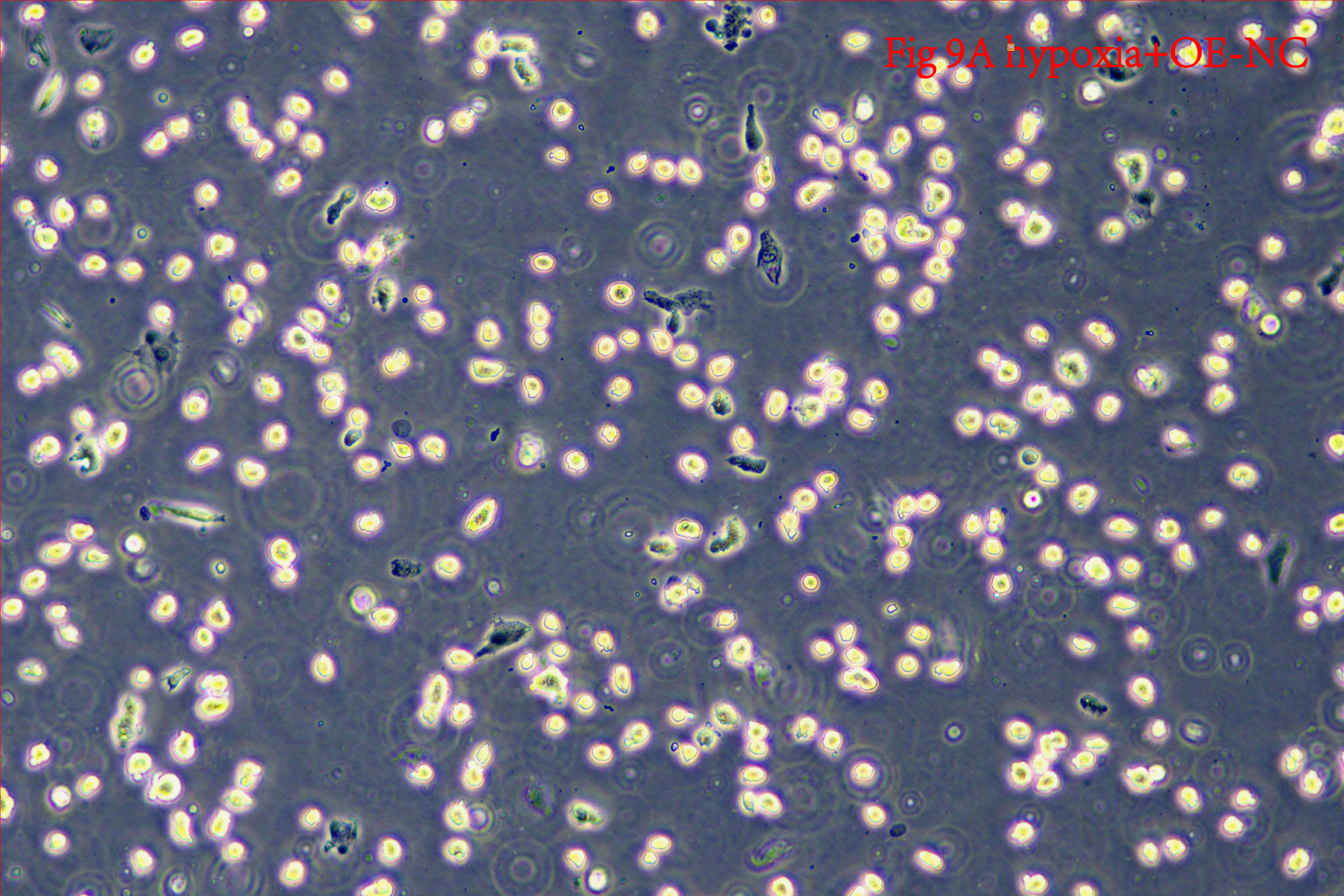

Fig 9A hypoxia+OE-Hpgd

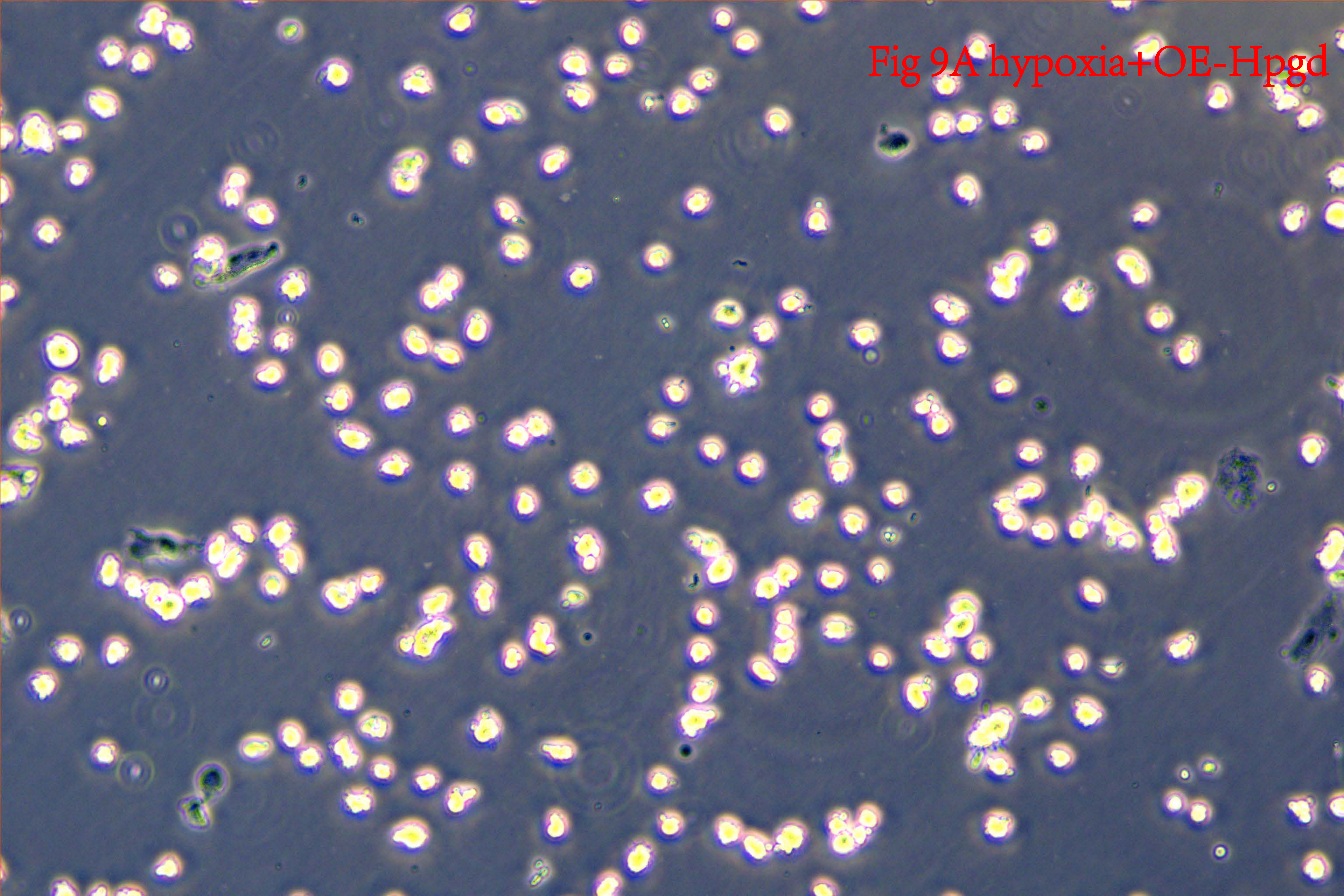

Fig 9C hypoxia+OE-NC

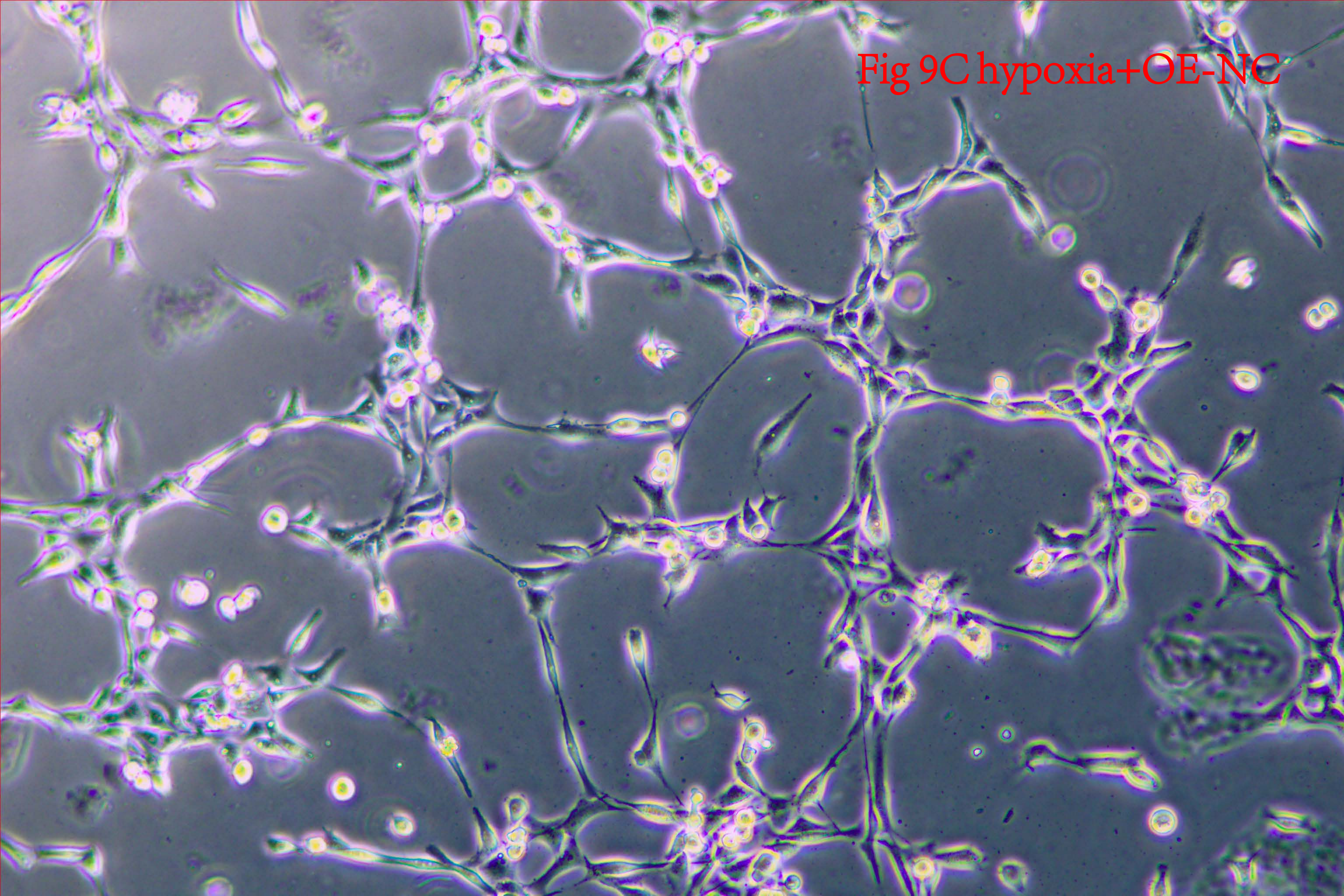

Fig 9C hypoxia+OE-Hpgd

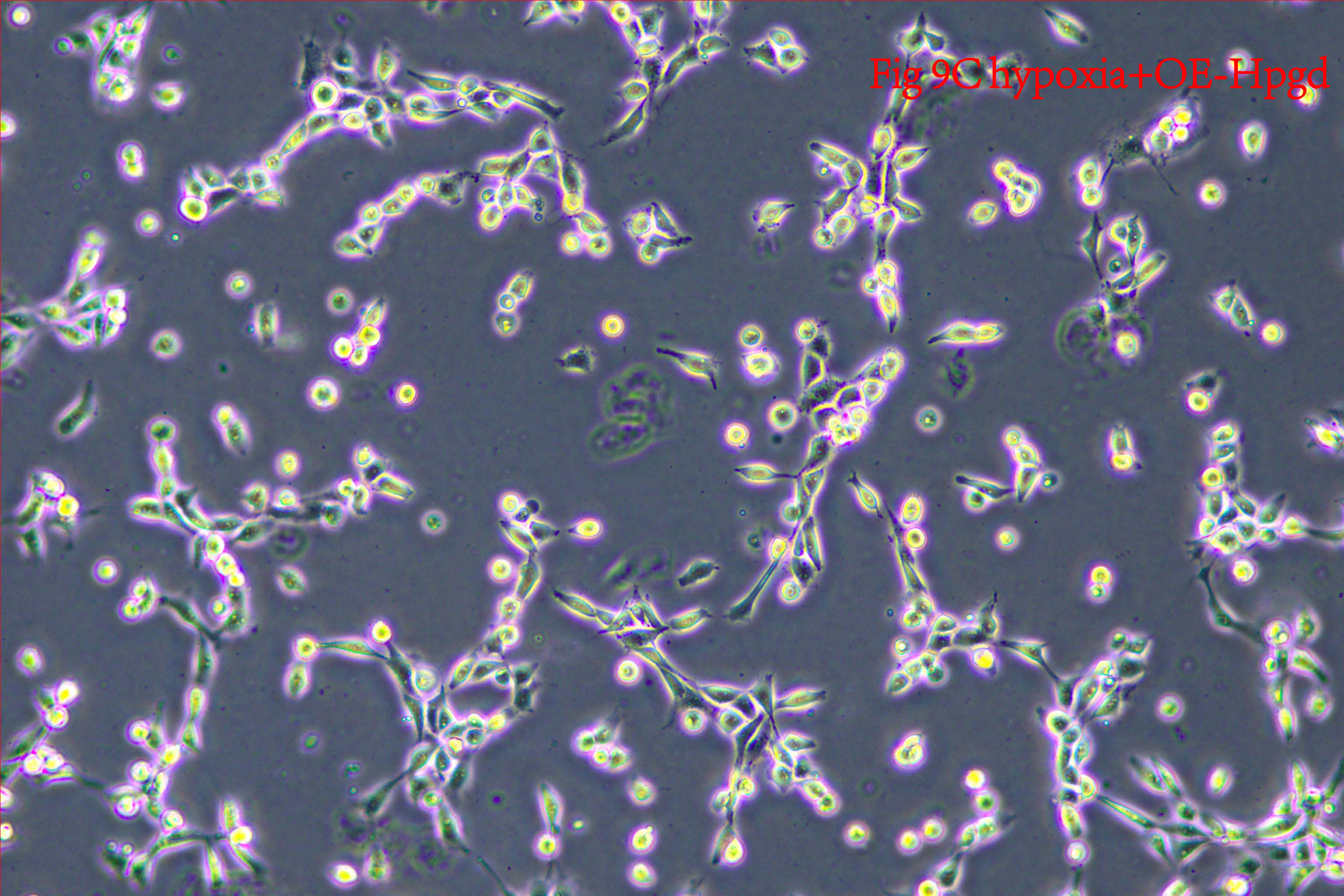

Supplement: Supplementary file 8 — Supplementary Material 8 [file 12890_2023_2401_MOESM8_ESM.pdf]
