# Supplementary material for: Hpgd affects the progression of hypoxic pulmonary hypertension by regulating vascular remodeling
Source: BMC Pulm Med. 2023 Apr 13;23:116. doi: 10.1186/s12890-023-02401-y (PMC10103477; doi:10.1186/s12890-023-02401-y)
Supplement: Supplementary file 6 — Supplementary Material 6 [file 12890_2023_2401_MOESM6_ESM.pdf]

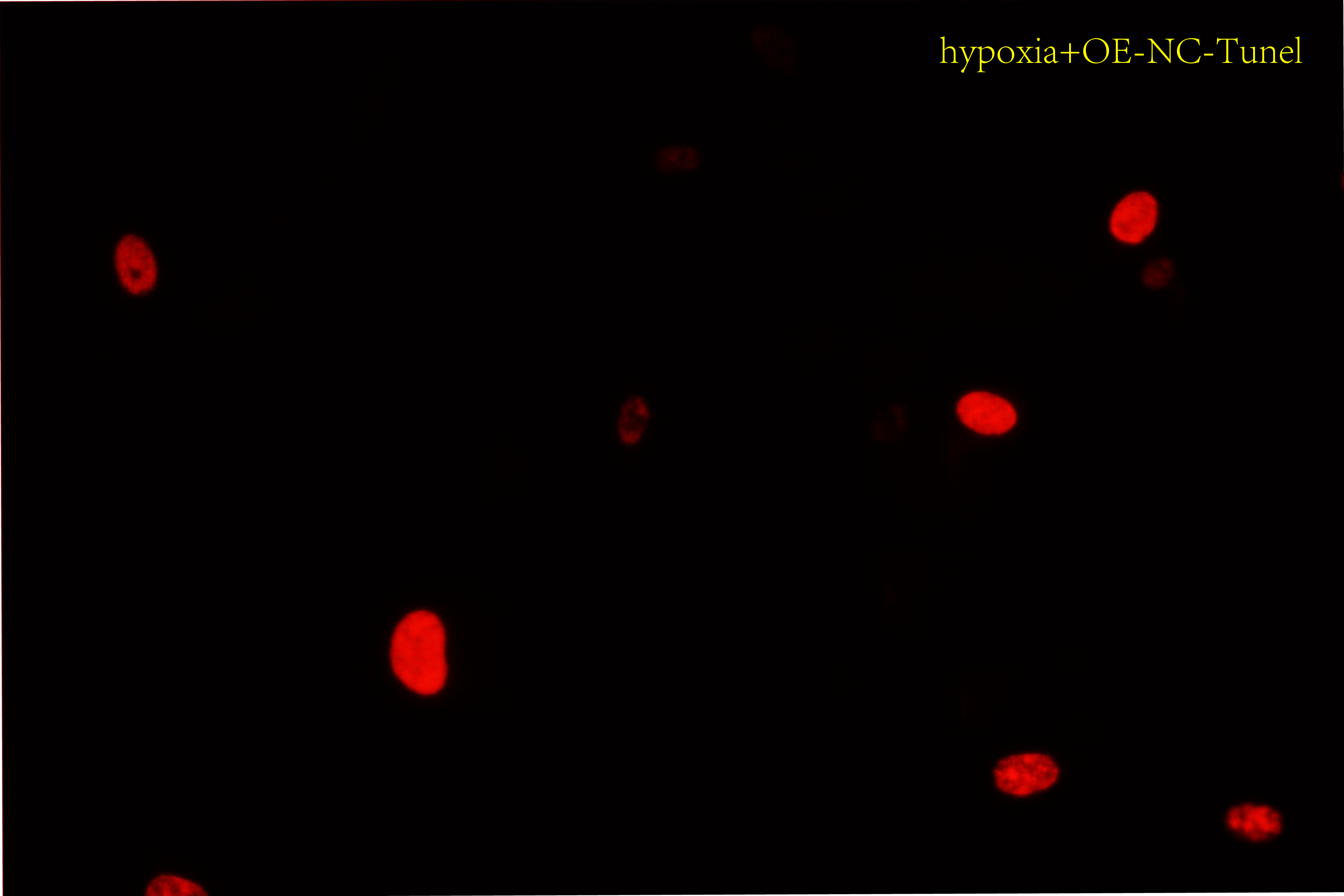
hypoxia+OE-NC-Tunel

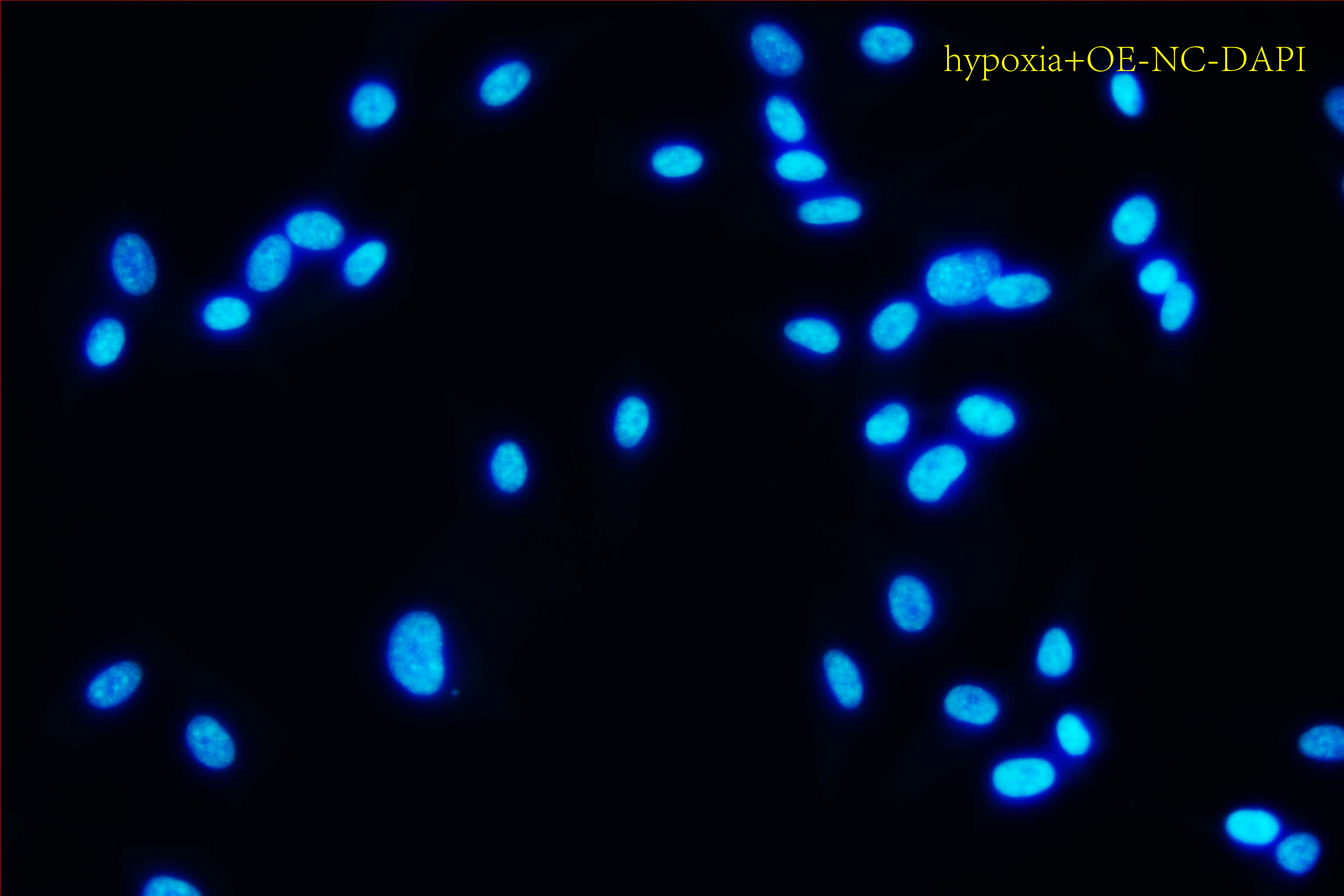
hypoxia+OE-NC-DAPI

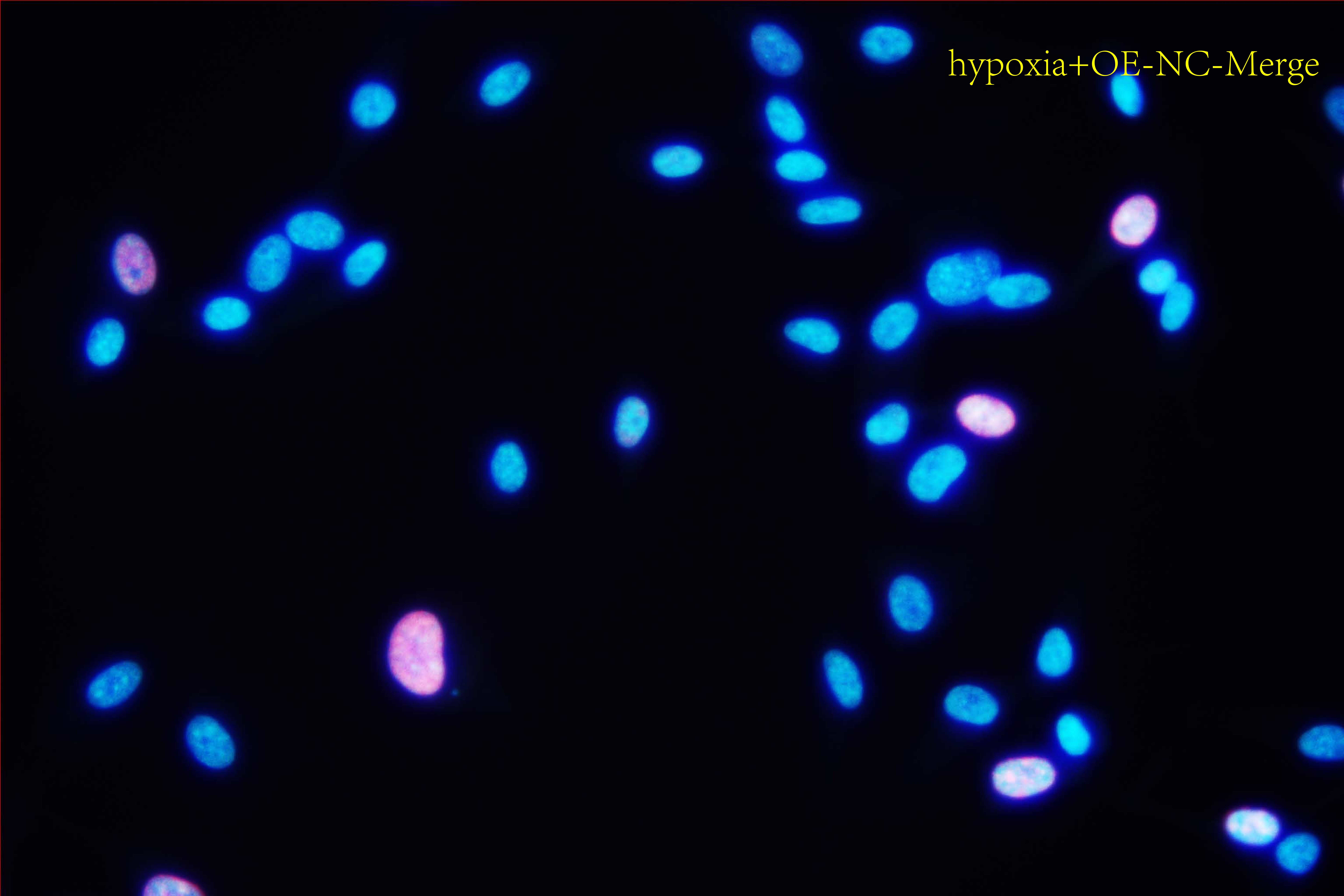
hypoxia+OE-NC-Merge

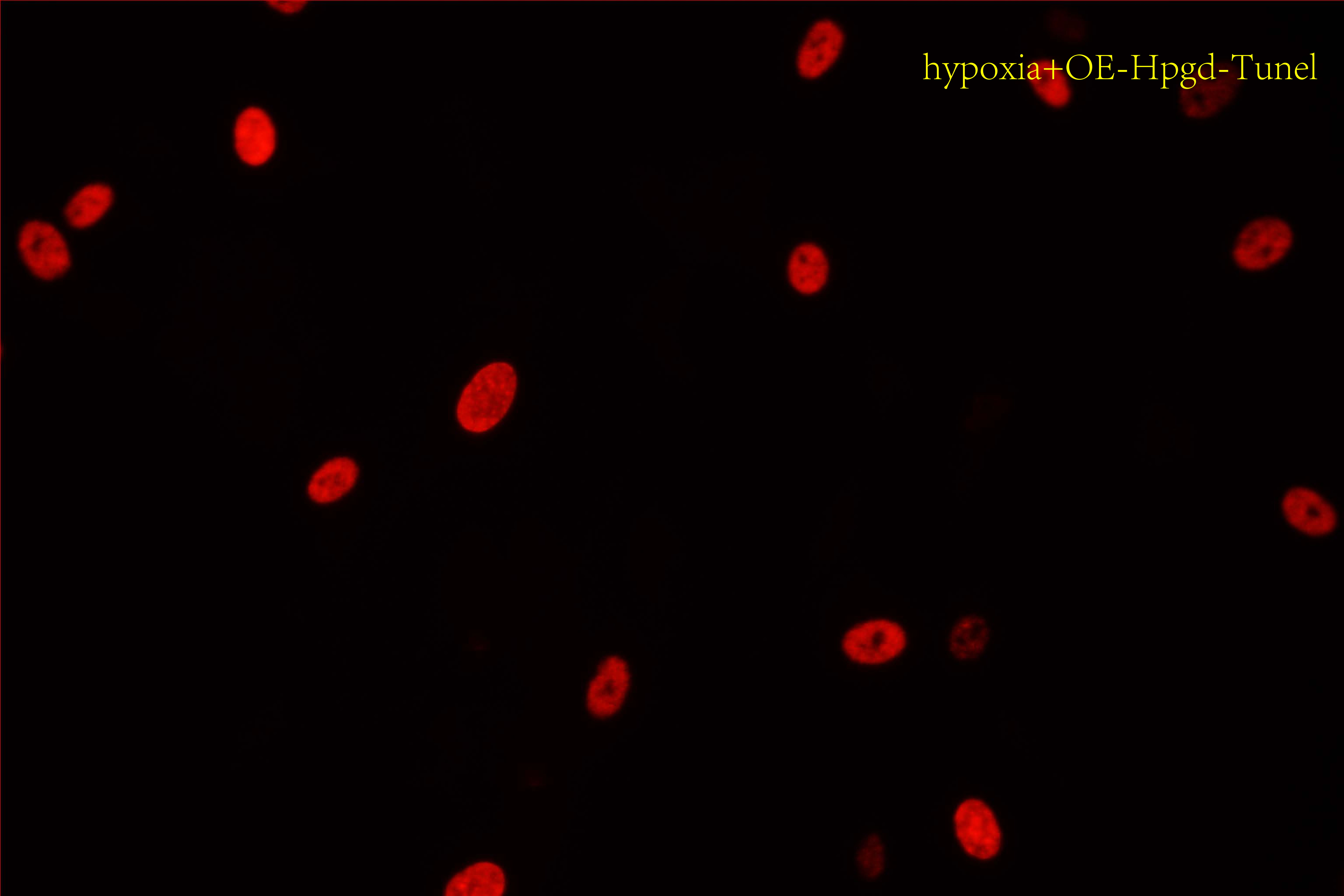
hypoxia+OE-Hpgd-Tunel

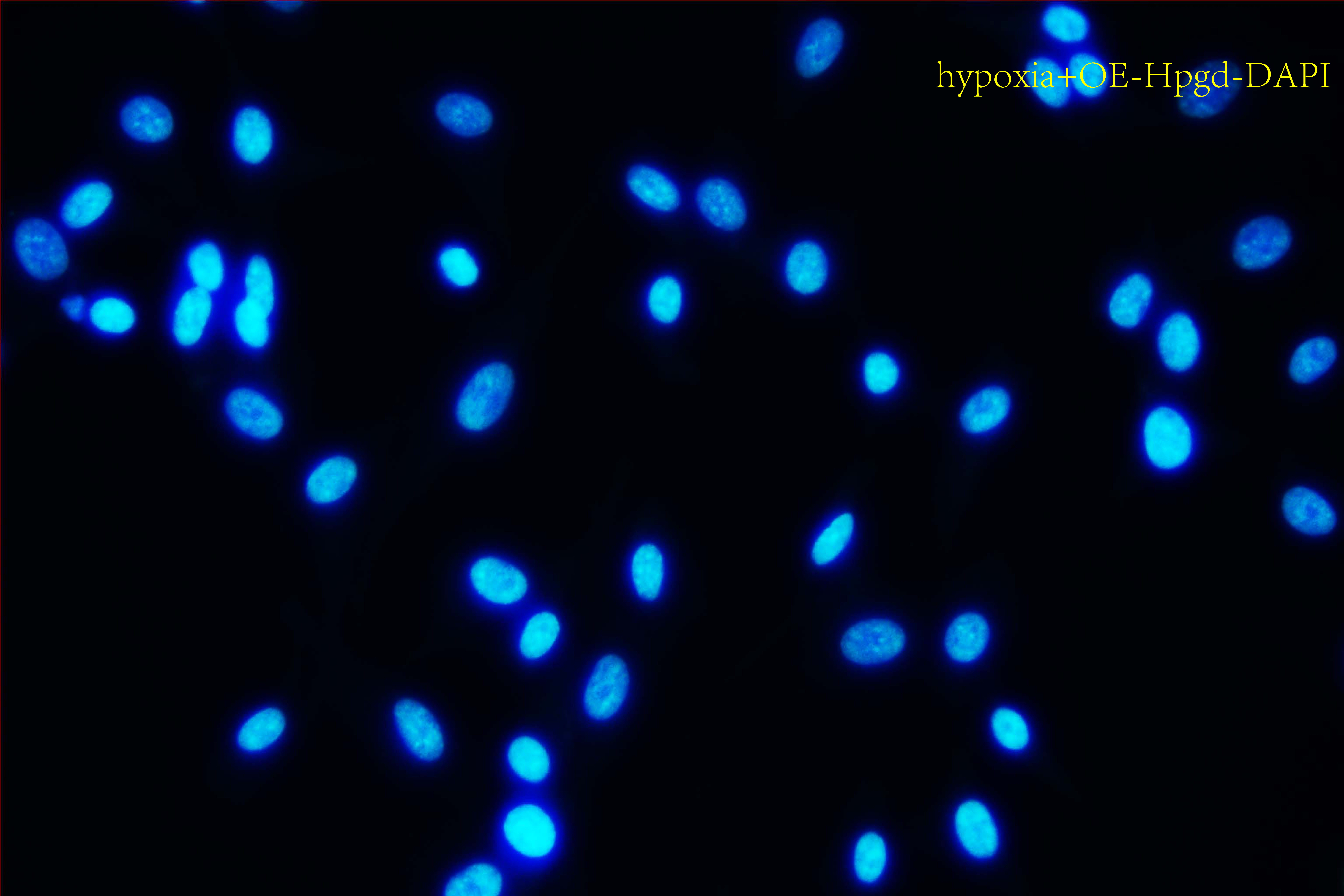
hypoxia+OE-Hpgd-DAPI

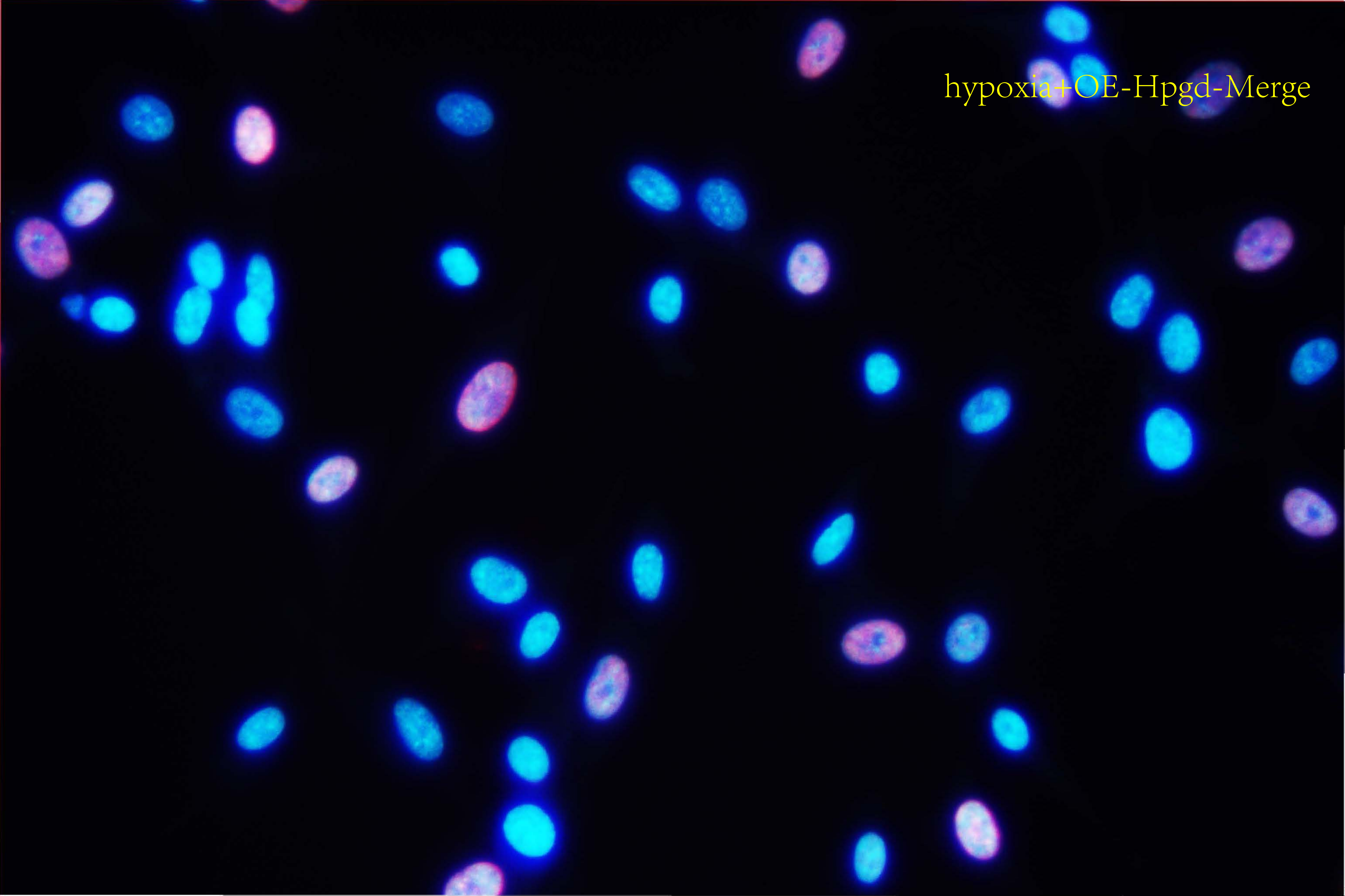
hypoxia+OE-Hpgd-Merge
